# Serotype distribution of invasive Pneumococcal disease in a tertiary children’s hospital in Vietnam

**DOI:** 10.1186/s12879-025-10672-5

**Published:** 2025-03-26

**Authors:** Hai Thien Do, Lam Van Nguyen, Nhung Trang Thi Nguyen, Ngoc Bich Thi Hoang, Dien Minh Tran, Hanh Phuong Nguyen

**Affiliations:** 1 Vietnam National Children’s Hospital, Hanoi, Vietnam; 2 Hanoi University of Public Health, Hanoi, Vietnam

**Keywords:** IPD, Children, Vietnam, Antimicrobial resistance, Pneumococcal serotypes, Clinical epidemiology

## Abstract

**Background:**

Invasive pneumococcal disease (IPD) is a leading cause of morbidity and mortality among children worldwide. However, this evidence from Vietnam is rare.

**Methods:**

This retrospective cross-sectional study was conducted at a tertiary children’s hospital in Vietnam by analysing data of all patients under 18 years old admitted the hospital for IPD from 2019 to 2022. *S. pneumoniae* isolates or DNA extract from blood or other normally sterile fluids were serotyped. Clinical characteristics, antibiotic susceptibility, serotype distribution, and patient outcomes were analyzed and reported.

**Results:**

Among total of 274 children with IPD identified, 232 children under 5 years old accounted for 84.7%. *S. pneumoniae* serotyping revealed 18 different serotypes, with 6A/B, 19A, 23F, 19F, and 14 being the most prevalent. The coverage rates for PCV7/PCV10, PCV13 and PCV20 were 66.0%, 83.0%, and 87.2%, respectively. Antibiotic resistance rates were high, with nearly 100% resistance to erythromycin and clindamycin, and a 75.3% resistance rate to third-generation cephalosporins. Amongst 24 death cases, 25% of the confirmed deaths attributed to serotype 19F.

**Conclusion:**

Paediatric IPD in Vietnam primarily affects children under 5 years old, with high rates of antibiotic resistance. The high pneumococcal conjugate vaccine (PCV) coverage emphasizes the need for universal PCV vaccination in children to enhance IPD prevention. Continued and enhanced surveillance of IPD is essential for better understanding and management. **Clinical trial number**: not applicable.

## Background

Pneumococcal infections can result in common childhood diseases including sinusitis and acute otitis media, as well as severe invasive diseases such as meningitis and septicemia [1]. These infections can be fatal, particularly in children from low- and middle-income countries (LMIC) where up to 20% of affected children die from septicemia and 50% from meningitis [2].

While the number of invasive pneumococcal disease (IPD) cases remains high in LMIC, high-income nations have experienced a significant decline in this prevalence [3, 4]. This disparity might be due to limited access to pneumococcal conjugate vaccines (PCV) and delays in integrating these vaccines into national immunization programs in LMIC. In contrast, high-income countries had an extensive utilization of PCVs [3, 4]. Not only has this vaccines diminished IPD rates, but it has also shifted the IDP prevalence towards non-vaccine serotypes [5, 6]. Therefore, the IPD situation in LMICs poses a major risk to community health.

Some studies from Vietnam have emphasized that *Streptococcus pneumoniae* is a significant bacterial agent causing meningitis and pneumonia [7, 8, 9]. Following the introduction of the *Haemophilus influenzae* type b vaccination into the national immunization program, reports indicate that *S. pneumoniae* has emerged as the primary cause of bacterial meningitis in children, with case fatality rate was 10% and up to 23% of survivors experiencing long-term neurological sequelae [10].

Although the prevalence of IPD remains high, Vietnam has not yet included PCVs in the national immunization program. The adoption of PCVs has been primarily restricted to private purchases and out-of-pocket expenditures, therefore limiting accessibility for the general population and presenting significant challenges for lower-income families. Moreover, data on the serotype distribution of *Streptococcus pneumoniae* in Vietnam, particularly in children, has been sparse. This lack of evidence affects decisions regarding vaccine coverage expansion in Vietnam and other LMICs.

To fill these knowledge gap, this study aims to analyze the clinical characteristics and serotype distribution of IPD in a tertiary children’s hospital in Vietnam from 2019 to 2022. The findings will serve as a valuable guide for vaccine design, recommendations, and strategies to reduce the overall impact of pneumococcal disease.

## Method

### Research design

This is a retrospective, descriptive, cross-sectional study.

### Setting

Vietnam National Children’s Hospital (VNCH), which is in Hanoi city, is the largest tertiary hospital dedicated to pediatric care in northern Vietnam. With 2,300 beds and 4,500–5,000 out-patients daily, VNCH serves a population of approximately 30 million people. The hospital’s comprehensive capabilities and specialized care for severe infectious diseases, including IPD, coupled with the high referral rate (72%) from other provinces, ensured a representative and diverse cohort for this study.

### Participants

Children under the age of 18 admitted to the VNCH for IPD between 2019 and 2022 were recruited for the study.

IPD is defined as the presence of *Streptococcus pneumoniae* bacteria isolated from a normally sterile site, or the detection of pneumococcal DNA in cerebrospinal fluid (CSF) or pleural fluid. Only cases with isolate or sample taken within 1 week of admission were included in this study. Patients without clinical presentations consistent with invasive diseases were excluded from the study.

### Data collection processes

Cases of IPD were identified through dual screening methods: microbiological review of culture and molecular diagnostic results, and examination of International Classification of Disease-10 diagnostic codes at discharged time related to IPD. Data collection was standardized using a unified case record form. All extracted data were entered into a study database using Kobotoolbox platform. For patients with multiple specimens during a single hospitalization period, only one specimen was included in the analysis. In cases where multiple positive samples were available, priority was given to cultured specimens if the clinical sample was retained and suitable for serotyping.

### Laboratory and serotyping methods

Blood specimens were cultured using the BioMérieux automated blood culture system or BD Bactec FX plus. Positive blood samples were cultured on 5% sheep blood agar medium. CSF was cultured directly onto blood agar medium. Following inoculation, the culture media were incubated in an incubator set to a temperature of 35–37⁰C with 5–7% CO_2_. Colonies isolated after culturing were identified using the VITEK 2 automatic system or using VITEK MS system (MALDI-TOF). Antibiotic susceptibility testing was performed by an automated system (VITEK 2 Compact, France). According to the validation study by Damien Dubois et al., VITEK MS demonstrated excellent discrimination between *S. pneumoniae* and other alpha-hemolytic streptococci, with a sensitivity of 99.1% and specificity of 100% [11]. Therefore, the VITEK MS system is considered reliable and highly accurate for use in clinical applications. The minimum inhibitory concentration (MIC) of antibiotics for *S. pneumoniae* was determined in accordance with the guidelines by the Clinical and Laboratory Standards Institute (2018 edition). The American Type Culture Collection (ATCC 49,619) strain of *S. pneumoniae* was used as controls during the susceptibility test.

All bacterial isolates from IPD patients hospitalized during the study period (2019–2022) were preserved at -70 °C in CRYOBANK microbial strain storage tube. After identifying the list of retrospective strains, the microbiology department retrieved these strains from frozen storage and performed two consecutive cultures on 5% sheep blood agar medium. Only viable strains after subculture were proceeded to serotyping.

In the case of body fluids where the detection of pneumococci was performed by molecular methods, two *S. pneumoniae* genes (*lytA* and *wzg*) were used for bacterial identification.

Serotypes were determined using CDC-recommended multiplex PCR assays capable of detecting 70 pneumococcal serotypes [12]. When PCR could not definitively distinguish between certain serotypes (e.g., 6 A vs. 6B, or 9 A vs. 9 V), these were grouped based on their inclusion in PCV vaccine coverage. Specifically, ambiguous results like 6A/B or 9 A/V were classified as PCV7-covered serotypes since both potential variants are targeted by the PCV7 vaccine.

### Data analysis

The study reported the percentages and 95 confidence intervals (CI) of each outcome variables. The Chi square test (χ2) or 2-tailed Fisher exact test were used to examine different amongst percents. Univariate logistic regressions were used to identify association between certain serotypes and the severity of disease. A p-value smaller than 0.05 was considered statistically significant.

We defined clinical syndromes of invasive diseases as follows: Meningitis was defined as *S. pneumoniae* identified (via culture/polymerase chain reaction [PCR]) in the CSF or *S. pneumoniae* cultured from blood with radiological and/or clinical features of meningitis. Pneumonia was defined as *S. pneumoniae* identified in pleural/empyema fluid or in blood, plus the presence of clinical symptoms and chest imaging (X-ray, ultrasound, or CT scanner) of pneumonia. Other invasive diseases (arthritis, mastoiditis, peritonitis, subcutaneous abscess) are defined as *S. pneumoniae* isolated from normally sterile fluids (synovial fluid, peritoneal fluid…) with the presence of corresponding clinical syndromes. Septicaemia without focus was diagnosed when no focal infection could be identified.

High-risk conditions for IPD include proven or presumptive CSF leak (cochlear implants, intracranial shunts), children born before 28 weeks of pregnancy) asplenia, nephrotic syndrome, congenital heart disease, chronic liver disease, malignancy, primary or acquired immunodeficiency or use of immunosuppressive therapy in the 90 days preceding IPD and Down syndrome [13]. The sequelae in survivors of meningitis were defined based on the clinician’s assessment at the time of discharge. The following conditions were defined as severe conditions: mechanical ventilation, septic shock, longer hospital stay, surgical interventions, and sequelae in survivors. A longer hospital stay is defined as exceeding the 75th percentile of the interquartile range (IQR) for the length of hospital stays observed in this study.

Data was analysed using Stata software version 18.0 (Stata Corp, College Station, Texas).

## Results

### Demographics of children with IPD

In total, there were 274 patients with IPD during the time of study; 127 (46.4%) were female and 147 (53.6%) were male. The median age was 15.0 months, with an IQR of 7.0 to 31.0 months. Among the patients, 232 (84.7%) were younger than 5 years old.

A seasonal trend was observed with 50.7% (*n* = 139) patients admitted to the hospital during the period from October to January each year, which corresponds to the winter season in Northern Vietnam.

The median length of hospital stays was 20 days (p25th-p75th, 12.0–29.0).

There were 57 (20.8%) patients with underlying diseases. The main underlying diseases were congenital heart disease (10 cases, 17.5%), biliary atresia (9 cases, 15.8%), CSF leakage (9 cases, 15.8%), nephrotic syndrome (5 cases, 8.8%), and epilepsy (4 cases, 7.0%).

### Clinical manifestations of IPD patients

Meningitis (59.9%, *n* = 164) was the most common manifestation, followed by bacteremia pneumonia (*n* = 55, 20.1%) and septicemia without focus (12.8%) (Table 1).

Other clinical manifestations include mastoiditis (12 cases), primary peritonitis (2 cases), primary peritonitis with pneumonia (1 case), osteomyelitis (1 case), spinal extradural abscess (1 case), and subcutaneous abscess (3 cases).

The distribution of clinical manifestations varied with age. The incidence of meningitis and pneumonia in children under 2 years of age is higher than in the rest of the age groups (*p* < 0.05).

**Table 1:** Characteristics of IPD in children

Age (months)	0–23	24–59	>=60	*p*
***Gender***	177	55	42	
Male (n, %)	93 (63.3%)	26 (17.7%)	28 (19.0%)	0.152
***Clinical manifestations***				
Meningitis (n, %)	110 (67.1%)	24 (14.6%)	30 (18.3%)	0.013
Bacteremia pneumonia (n, %)	30 (54.5%)	18 (32.7%)	7 (12.7%)	0.039
Septicemia without focus (n, %)	24 (68.6%)	09 (25.7%)	02 (5.7%)	0.205
Other (n, %)	13 (65.0%)	4 (20.0%)	3 (15.0%)	1.000
***Complications***				
Neurological complications	73 (83.0%)	8 (9.1%)	7 (7.9%)	< 0.001
Respiratory complications	16 (59.3%)	8 (29.6%)	3 (11.1%)	0.804
***Disease severity***				
Septic shock	28 (73.7%)	08 (21.0%)	02 (5.3%)	0.17
MODS	02 (66.7%)	01 (33.3%)	00 (00%)	0.732
Mechanical ventilation	59 (75.6%)	11 (14.1%)	8 (10.3%)	0.005
Surgical intervention	68 (78.2%)	13 (14.9%)	6 (6.9%)	0.004
Sequelae in meningitis	23 (82.1%)	2 (7.1%)	3 (10.7%)	0.2

Among 150 children with meningitis had CT scanner or MRI of the brain, 86 cases showed abnormal findings. They include hydrocephalus (47 cases, 55.3%), subdural empyema (30 cases, 35.3%), cerebrovascular complication (16 cases, 18.8%) (hemorrhage, venous thrombosis, cerebral infarction etc.), brain abscess (2 cases, 2.4%), cerebral parenchyma lesions (25 cases, 29.4%), and diffuse lesions (14 cases, 16.3%) (not mutually excluding). Of the 14 cases that did not have CT scanner or MRI of the brain, 12 were discharged with no sequelae so we considered those cases as meningitis without complications, and 2 were admitted to the hospital in conditions so severe it was impossible to obtain CT scan or MRI of the brain, so we classified them as neurological complications. Thus, the number of cases of meningitis with complications is 88 cases (53.7%). Concerning age, a higher proportion of patients under the age of 2 with meningitis exhibited neurological problems compared to patients in other age groups (*p* < 0.05) (Table 1).

Twenty-seven children were diagnosed with complications of pneumonia, which included pleural effusion/empyema in 22 cases, necrotizing pneumonia with empyema in 2 cases, acute respiratory distress syndrome (ARDS) in 2 cases, and pneumonia with hemolytic uremic syndrome in 1 case.

Eleven patients (4.0%) experienced multiple viral infections at the same time, comprising 5 cases of influenza, 3 cases of adenovirus, 1 case of rotavirus, 1 case of EV71, and 1 case of COVID-19.

### Antimicrobial susceptibility testing

The antibiotic susceptibility of the 160 isolates was assessed and the results are presented in Table 2. The *S. pneumoniae* isolates were nearly 100% resistant to Erythromycin and Clindamycin. A very high rate of beta-lactam non-susceptible antimicrobials with meningitis breakpoint (Penicillin 94.7%, Ceftriaxone 74.5%, Cefotaxime 75.3%) was also observed. All isolates were susceptible to levofloxacin, vancomycin, and linezolid.

**Table 2:** Susceptibility of *S. pneumoniae* isolates to antimicrobial agents

	Non-susceptible	Total isolates
Chloramphenicol	17 (20.5%)	83
Clindamycin	116 (95.9%)	121
Erythromycin	120 (98.4%)	122
Penicillin		
Meningitis	54 (94.7%)	57
Non-meningitis	34 (42.0%)	81
Ceftriaxone		
Meningitis	70 (74.5%)	94
Non-meningitis	42 (34.1%)	123
Cefotaxime		
Meningitis	70 (75.3%)	93
Non-meningitis	72 (47.4%)	152
Vancomycin	0 (0.0%)	160
Levofloxacin	0 (0.0%)	159
Linezolid	0 (0.0%)	68

In this study, 92 *S. pneumoniae* isolates with antimicrobial susceptibility testing were serotyped. The nonsusceptibility patterns of the most prevalent serotypes were presented in Table 3. While serotype 6A/B and 14 showed high nonsusceptibility to chloramphenicol and penicillin (with non-meningitis break point), serotypes 19A and 23F remained highly susceptible to this antibiotic. Serotype 19F exhibited the highest nonsusceptibility rates to both ceftriaxone and cefotaxime (with non-meningitis break point).

**Table 3:** Antimicrobial nonsusceptibility of most prevalent *S. pneumoniae* serotypes

	6A/B (*n* = 14)	14 (*n* = 10)	19A (*n* = 16)	19F (*n* = 17)	23F (*n* = 18)
	% Non-susceptibility				
Chloramphenicol	54.5	33.3	0	14.3	0
Clindamycin	91.7	100	100	90	100
Erythromycin	92.3	100	100	100	100
Penicillin					
Meningitis	100	100	100	100	100
Non-meningitis	50.0	50.0	0	42.9	33.3
Ceftriaxone					
Meningitis	81.8	75	66.7	90	100
Non-meningitis	16.7	50	30.8	66.7	11.1
Cefotaxime					
Meningitis	81.8	75.0	83.3	80.0	100
Non-meningitis	21.4	55.6	43.8	70.6	55.6

### Serotype distribution and vaccine coverage

A total of 81 isolates (57.4%) and 60 fluid samples (42.6%) that tested PCR-positive for *S. pneumoniae* were sent to the laboratory for serotyping. Blood was the source for 31.9% (45) of these samples, while 68.1% were from CSF.

In this study, we identified the presence of 18 serotypes. The most prevalent serotypes identified were 6A/B (*n* = 29, 20.57%), followed by 19A (*n* = 24, 17.02%), 23F (*n* = 22, 15.6%), 19F (*n* = 20, 14.18%), and 14 (*n* = 14, 9.93%). Other serotypes with lower prevalence rates include 15A/F, 15B/C, and 9A/V, each with 6 isolates (4.26%), and serotypes 23A and 4, with 2 (1.42%) and 1 (0.71%) isolate, respectively. Ambiguity in serotyping was noted in a few instances – 12F/A/B/44/46, 33F/A/37, and 18A/B/C/F, each with one isolate. There are nine samples (6.38%) for which the serotypes could not be determined.

**Fig. 1:**
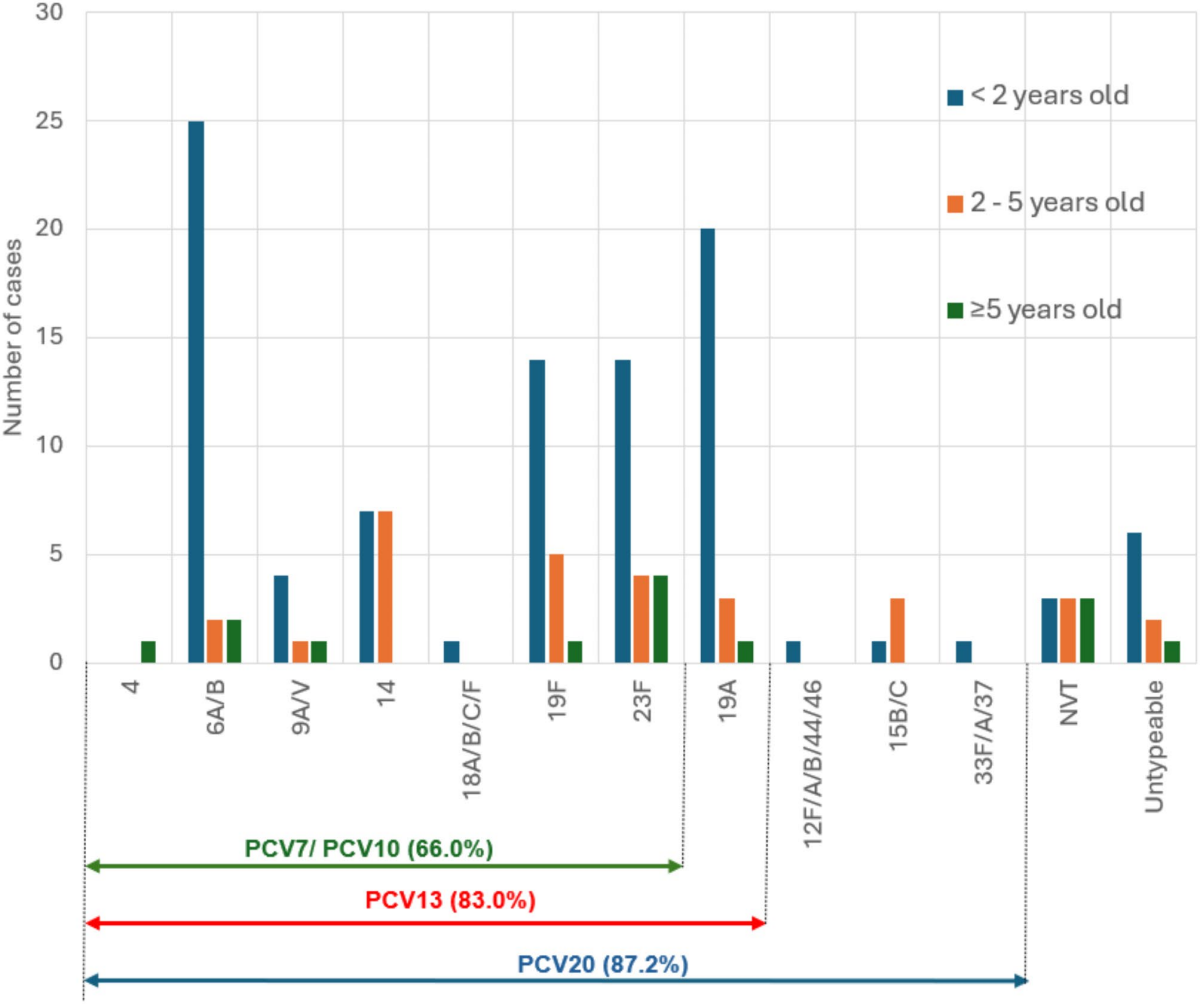
Serotype distribution of IPD cases by age group and PCV coverage (NVT = non-vaccine serotype) *Note: When serotypes could not be definitively distinguished, they were classified based on PCV coverage of their possible variants*

The coverage rates of PCV-7/PCV-10, PCV-13 and PCV-20 were 66.0%, 83.0% and 87.2%, respectively. (Fig. 1)

The distribution of serotypes differed among different age groups, as seen in Fig. 1. Within each age group, different serotypes showed varying prevalence. Serotype 6A/B was the most common among children under 2 years old, accounting for 25.5% of cases in this age group. In the 2 to 5-year-old age group, serotype 14 was the prevailing strain, making up 24.1% of cases. Among children aged 5 years and older, serotype 23F had the greatest incidence rate, at 28.6%.

Further analysis of serotype distribution specifically in children under five years old (*n* = 127) revealed that the five most prevalent serotypes were 6A/B (27 cases, 21.26%), 19A (23 cases, 18.11%), 19F (19 cases, 14.96%), 23F (18 cases, 14.17%), and 14 (14 cases, 11.02%), which closely mirrored the overall serotype distribution observed in the total study population.

**Fig. 2:**
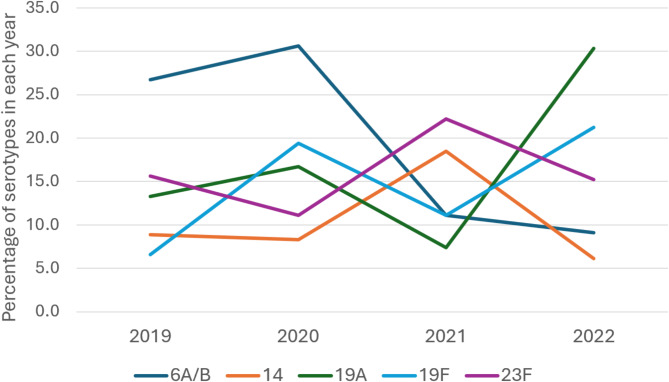
Annual percentage distribution of serotypes 6A/B, 14, 19A, 19F, and 23F from 2019 to 2022

The distribution patterns of serotypes 14, 19F, and 23F showed fluctuations across the study period without significant trends. However, notable changes were observed for 6A/B and 19A. The proportion of serotype 6A/B demonstrated a marked decrease from 26.1% in 2019 to 9.1% in 2022. Serotype 19A showed an increase from 13.3 to 30.3% in 2020. (Fig. 2)

**Fig. 3:**
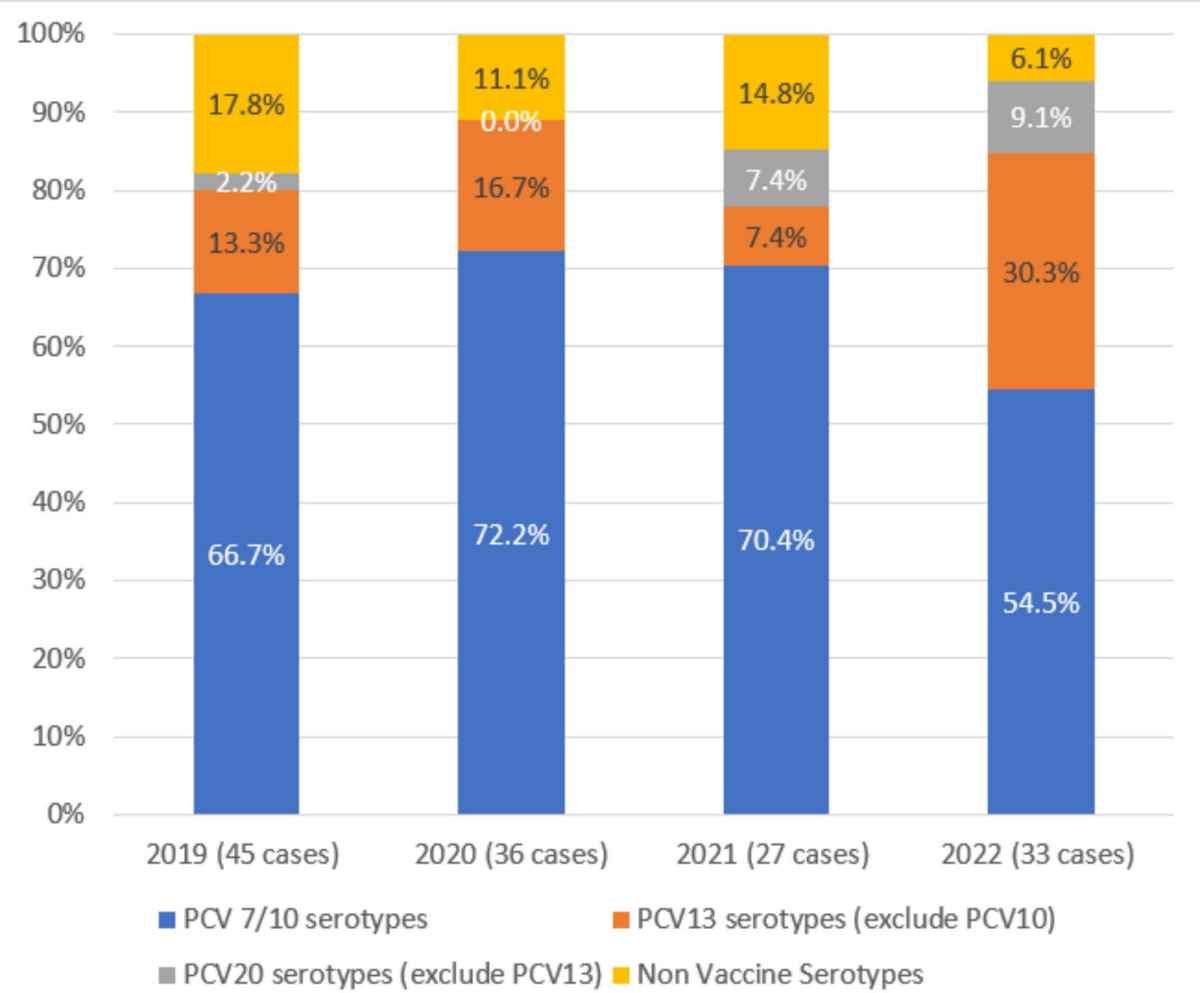
Yearly IPD serotype data from 2019 to 2022, grouped by vaccine coverage

In our study, the difference between PCV13 and PCV7/PCV10 coverage was attributed to serotype 19A prevalence (Fig. 1), as serotype 3 was not present in the study and serotype 6A/B was classified under PCV7 coverage. The prevalence of PCV7/10 coverage decreased from 66.7 to 54.5% across four years (Fig. 3). In contrast, additional serotypes covered by PCV13 (19A) showed an overall increasing pattern, rising from 13.3 to 30.3% observed in 2022. Serotypes unique to PCV20 also demonstrated an upward trend, increasing from 2.2 to 9.1%. Meanwhile, non-vaccine serotypes (NVT) showed a consistent decline from 17.8 to 6.1% over the study period. The total coverage of vaccine serotypes (PCV7/10, PCV13, and PCV20 combined) increased from 82.2% in 2019 to 93.9% in 2022.

### Association between Pneumococcal serotypes and mortality

The overall mortality rate in this study was 8.8% (24 deaths). For children under 5 years old (*n* = 232), mortality reached 9.91% (23 deaths). Among fatal cases, eight (33.3%) had underlying diseases. Of the 14 cases (58.3%) with serotype identification, there were six cases of serotype 19F, three cases of serotype 23F, two cases each of serotypes 19A and 6A/B, and one case of serotype 9 A/V.

Statistical analysis revealed a significant association between serotype 19F and increased mortality risk (Table 4). This serotype was also associated with higher rates of mechanical ventilation and development of septic shock, although these associations were not statistically significant. No significant correlations were identified between serotype 19F and longer hospital stays, sequelae in survivors, or the necessity for surgical intervention.

**Table 4:** Association between serotype 19F and mortality and severe conditions

	Death	Mechanical ventilation	Septic shock	Longer hospital stays	Surgical intervention	Sequelae in survivors
OR (95%CI)	6,054 (1,739 − 21,063)	2.667 (0.042–0.910)	2.812 (0.056–0.927)	1.244 (0.437–3.534)	1.160 (0.791 − 0.385)	0.785 (0.761 − 0.164)
p	0.006	0.604	0.089	0.784	0.777	1.000

## Discussion

This study highlighted the burden of invasive pneumococcal disease (IPD) among children in Vietnam from 2019 to 2022. The data reveal a higher prevalence of IPD among males (53.6%) and primarily in children under 5 years of age (84.7%), as mentioned in another study [14]. The findings emphasized that meningitis was the most common clinical manifestation of IPD, especially in children less than 2 years of age. More than half of meningitis cases (53.7%) had neurological complications. This high rate of meningitis complications might be due to delays in diagnosis and treatment, limited access to healthcare services and antibiotic resistance in Vietnam.

Our findings confirmed change of trend and serotype distribution of IPD over time. The study revealed a predominant prevalence of serogroups 6A/B, 23F, 19A, 19F, and 14, with the PCV13-serotypes accounted for 82.5% of the total of the valid samples. This aligned with patterns observed in other LMICs with low PCV uptake, such as China, Serbia, and Thailand, where vaccine-targeted serotypes remain dominant [15, 16, 17]. For example, PCV13 serotypes accounted for 80.7% of pediatric IPD cases in China [15], while serotypes 19F, 14, and 6B dominated in Serbia [16] and Thailand [17].

In Vietnam (2015–2016), major *S. pneumoniae* serotypes detected among pediatric acute respiratory infections cases were 6A/B (35.9%), 19F (23.7%) and 23F (12.7%) [18]. This similarity between invasive and non-invasive diseases highlighted the persistent circulation of these strains in settings with limited immunization coverage.

Prior to the introduction of PCV, seven serotypes (1, 5, 6A, 6B, 14, 19F, 23F) were the most common cause of IPD among young children worldwide [19]. The absence of serotypes 1 and 5 in our study may reflect regional epidemiological differences or shifts driven by early vaccine introduction dynamics.

Notably, 6A/B - previously the fourth most common serotype in Vietnam prior to the introduction of PCV-7 in 2010 [20] - emerged as the dominant type in our study, likely reflecting our focus on children under two years. Concurrently, the rising prevalence of serotype 19A (22.5% in our study vs. 0.4% pre-PCV [20]) and the decline of 6A/B suggest early signs of serotype replacement, a phenomenon increasingly observed in countries with expanding PCV programs [4, 21]. For example, Japan documented rapid declines in PCV7 serotypes (from 73.3 to 14.7%) and concurrent increases in non-vaccine serotypes like 19A and 15A were documented as PCV7 coverage rose from < 10% to 80–90% between 2010 and 2012 [21].

In high-income countries, serotype replacement has led to diverse non-vaccine types (e.g., 8, 9N, 15A), reducing the projected benefits of expanding vaccine valency compared to earlier PCVs [22]. In Vietnam, however, staggered PCV7 implementation and variable coverage may have delayed this transition, explaining the continued dominance of PCV13 serotypes. Although these changes occurred during the COVID-19 pandemic (2019–2022), we found no conclusive evidence linking them directly to pandemic-related disruptions.

In our study, serotype 19F had the highest fatality rate (25% of deaths). This aligns with findings from a surveillance study [23] that demonstrated serotype 19F’s significant association with increased 30-day and 12-month mortality in IPD patients in England. The high mortality risk may be compounded by 19F’s concerning antibiotic resistance profile, with analysis showing the highest rates of non-susceptibility using non-meningitis breakpoints, while meningitis breakpoints revealed 100% resistance to penicillin, 90% to ceftriaxone, and 80% to cefotaxime. This resistance pattern is particularly concerning in Vietnam, where previous studies have documented high prevalence of multi-drug resistant 19F strains in paediatric pneumococcal carriage [24]. Given these intersecting challenges, incorporating PCV into Vietnam’s national immunization program could significantly impact both clinical outcomes and antimicrobial resistance patterns.

There are several limitations in this study. First, the retrospective nature may have introduced selection bias, and the quality of recorded data can vary. The hospital-based sample size limits the generalizability of our findings, although our patient population represents multiple regions across Vietnam. Technical limitations include the serotyping success rate of 57.4% (131 samples were of insufficient quality for analysis), which may affect the comprehensiveness of serotype distribution data. MALDI-TOF MS Vitek, which was used in our study, is usually considered a rapid and accurate method for identification of microorganisms, but has limitations, particularly in discriminating between *Streptococcus pneumoniae* and closely related viridans group streptococci. It has 100% specificity [11, 25], although studies have shown minimal misidentification rates - with only 1 out of 116 nonpneumococcal isolates (< 1%) being misidentified as *S. pneumoniae* [25]. Therefore, our results should be interpreted within this technical context.

## Conclusion

This is one of the initial paediatric IPD studies in Vietnam to contribute baseline data on clinical patterns and serotype distribution. Paediatric IPD in Vietnam primarily affects children under 5 years, with high antibiotic resistance rates. Serotype 6A/B, 19A, 23F, 19F, and 14 are the most prevalent ones. Future research needs to be conducted to validate these findings and establish more representative national patterns of disease burden and serotype distribution. The study also recommends PCV for all children to enhance IPD prevention efforts.

## Data Availability

The data utilized in this research has been securely stored in the Vietnam National Children’s Hospital. In accordance with Vietnamese regulations regarding data privacy and protection, we cannot share the original datasets outside our authorized research team. These regulations ensure the confidentiality and integrity of sensitive information, adhering to legal and ethical standards. We are committed to maintaining compliance with these regulations while also providing summaries and analyzed results that do not disclose any original data.

## Abbreviations

IPD: Invasive pneumococcal disease
PCV: Pneumococcal conjugate vaccine
LMICs: Low- and middle-income countries
CSF: Cerebrospinal fluid
MIC: Minimum inhibitory concentration
MODS: Multiple organ dysfunction syndrome
IQR: Interquartile range
